# Histological response to platelet-rich plasma added to polypropylene mesh implemented in rabbits

**DOI:** 10.1590/S1677-5538.IBJU.2015.0319

**Published:** 2016

**Authors:** Oscar Rubini Ávila, Natália Gomes Parizzi, Ana Paula Mayumi Souza, Dayane Silvestre Botini, João Ytimura Alves, Silvio Henrique Maia Almeida

**Affiliations:** 1 Departamento de Cirurgia, Universidade do Oeste Paulista, Presidente Prudente, São Paulo, Brasil;; 2 Departamento de Cirurgia, Universidade Estadual de Londrina, Londrina, Paraná, Brasil

**Keywords:** Histological Techniques, Rats, Blood Platelets

## Abstract

**Introduction:**

The platelet-rich plasma (PRP) is part of a set of biotechnologies, providing some growth factors that promote repair of different tissues. The polypropylene meshes (PPM) are applied in the correction of abdominal defects, pelvic floor and urinary incontinence, however, they induce many significant complications, as a result of an inappropriate inflammatory response.

**Purpose:**

To investigate the changes caused by PRP associated with the implantation of PPM in the abdomen of female rabbits, in the production of collagen I and III and the inflammatory infiltrate (ININ).

**Materials and Methods:**

We performed implant meshes with and without PRP in adult rabbits (n=30) and euthanasia at 7, 30 and 90 days. Two plates were prepared from each animal and analyzed in five different fields. The ININ was evaluated by quantification of inflammatory cells using hematoxylin-eosin and the collagen by Sirius red method. The results were analyzed applying the Wilcoxon, Kruskal-Wallis, Junckheere and Friedmann tests.

**Results:**

There was a significant difference in the number of inflammatory cells between the groups with and without PRP (p=0.01) at 90 days. There was increased production of collagen I, III and total with the use of PRP, at seven days.

**Conclusion:**

The PPM coating with PRP was associated with increased ININ at the implant area, and an increasing trend during the process of tissue repair. The PPM coated with PRP was related to increased concentration of collagen I, collagen III and the concentration of total collagen increased after seven days of implantation.

## INTRODUCTION

Synthetic mesh, of varying compositions, has been widely used for several years in the repair of hernias and in Urogynecology. However, its diverse properties and the inflammatory reaction caused by synthetic materials are still being studied (1–3).

Using synthetic mesh to repair abdominal hernias and urinary incontinence represents the largest group of implants in modern medicine, with about 1 million surgeries performed worldwide (4). Despite this fact, few randomized, controlled trials have evaluated the effects of the coated mesh during host incorporation.

Adverse results from the implant, including extrusion, erosion, and adhesion, can be related to abnormalities in the healing process (5). Baessler et al. showed erosion rates of 26% to 38% of dyspareunia using the polypropylene mesh in genital prolapse repair (6). The host's reaction to the implant is related to the type of material used; for example, an autologous fascia promotes lower inflammation and produces less collagen (7). Various protocols tested bioactive substances that act on inflammation and accelerate the repair process (8, 9). However, the complexity of the implant-host interaction impedes their use and causes controversy.

The platelet-rich plasma (PRP) definition is a plasma sample with a higher concentration of platelets, on average 2-3 times more, and is associated with clotting factors when compared to the peripheral blood (10). The potential therapeutic effect of PRP is the ability to promote tissue regeneration by releasing growth factors (CF) present in platelet alpha granules. These granules release growth factors soon after clot formation (11), and the proteins contained in it have a strong influence on reparative phenomena of wounds. The proteins include PDGF (“platelet derived growth factor”), TGF-Beta (“transforming growth factor”), and IGF (“insulin-like growth factor”), which exerts chemotactic, mitogenic, and angiogenic activity, which influences the inflammatory process. Some proteins released by platelets are absent in chronic wounds, emphasizing the role of these substances in tissue repair (12).

The PRP was chosen to study the acceleration of the repair process because it is a source of many growth factors (GF). It is already widely used in other medical fields (dermatology, orthopedics, dentistry and plastic surgery) and is widely available at a low cost (12, 13). The objective of this study was to examine the inflammatory infiltrate (ININ) response and collagen production induced by the implantation of polypropylene mesh in two groups, with or without PRP, using an animal model.

## MATERIALS AND METHODS

The Institutional Animal Care Utilization Committee approved the study. Thirty white New Zealand adult rabbits were submitted to sub aponeurotic implant of polypropylene mesh 1.0 x 1.0cm fragments (with pores of the 1500 microns).

The specimens were anesthetized with intramuscular Ketamine and Xylazine. The PRP gel was obtained after removal of 10mL of blood by cardiac puncture, and immediately taken to the laboratory for preparation using the protocol described by Anitua et al. (14).

**Figure 1:**
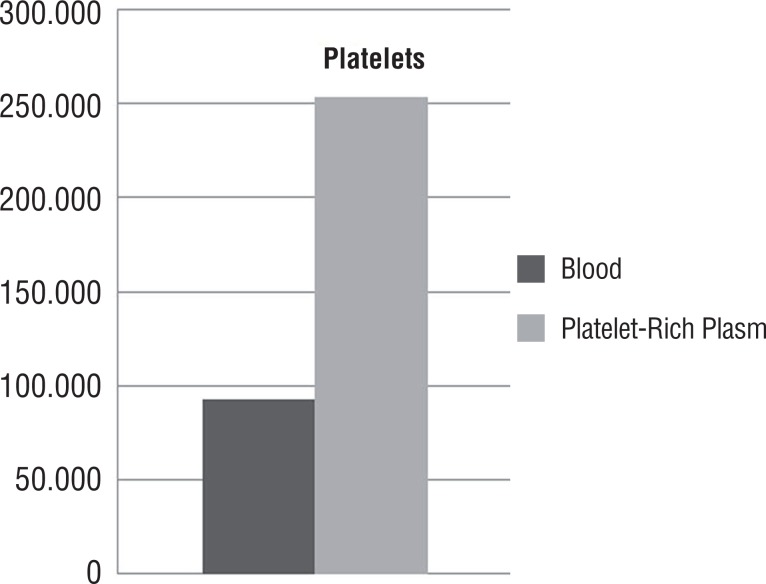
Mean platelet concentration in the blood compared to the centrifuged platelet-rich plasma (PRP).

Platelet counts in plasma of 25% of the samples chosen randomly after the process of preparation of PRP gel were performed to confirm the increase in the number of platelets. The gel count presented on average three times the platelet count than the peripheral blood (Figure-1).

A one-centimeter abdominal incision was performed, and the implant was accomplished without fixation to prevent tissue reactions. The mesh was implanted in a standardized manner, between the hypodermis and the fascia of the abdominal muscles. The animals were divided into 2 groups (15 each): mesh or PRP covering the mesh. Benzathine Penicillin was the antibiotic of choice.

The animals were euthanized after 7, 30 and 90 days after the implant 95 animals each from each group, with and without PRP.

All animals were anesthetized before the lethal injection. It was then removed from the block comprising the implant site interesting the skin, subcutaneous, mesh and muscle aponeurosis. At each time point, the wounds were harvested, and their histologic features were assessed in paraffin-embedded sections using hematoxylin & eosin and picro-sirius stains at a magnification of 10 X 40.

One pathologist was blinded to the mesh type and time from wounding evaluated all specimens. The slides were scanned under an Olympus microscope and a 3CCD pro-series digital camera; the capture and analysis program used was Image Pro-Plus (Media Cybernetics, Silver Spring, MD). The collagen type I, III, and total were assessed using polarized light (Sirius red), by computer morphometry (14) (density per micra^2^), while inflammatory infiltrate (H & E) were counted in different fields (400x). Four fragments of the material were placed on each slide.

For statistical studies were performed, including the Wilcoxon test (differences between the PRP groups and collagen types), the Kruskal-Wallis test (difference between point times), the Jonckheere trend test, and the Friedman test (differences inside groups). All statistical analysis was carried out using the SPSS version 13.0 data analysis system. P–values <0.05 were considered to be statistically significant for all comparisons.

## RESULTS

**Figure 2:**
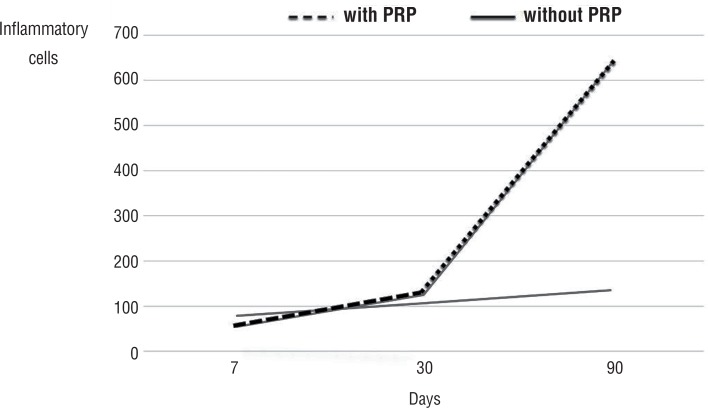
Curves of inflammatory infiltrate by time, accordingly with or without PRP.

Figure-2 shows the average sum of ININ per microscopic field to the rabbits euthanized at 7, 30, and 90 days comparing the group with or without PRP. The Jonckheere Test showed an increasing trend in the number of inflammatory cells into the euthanasia (p=0.027) in both groups.

**Figure 3:**
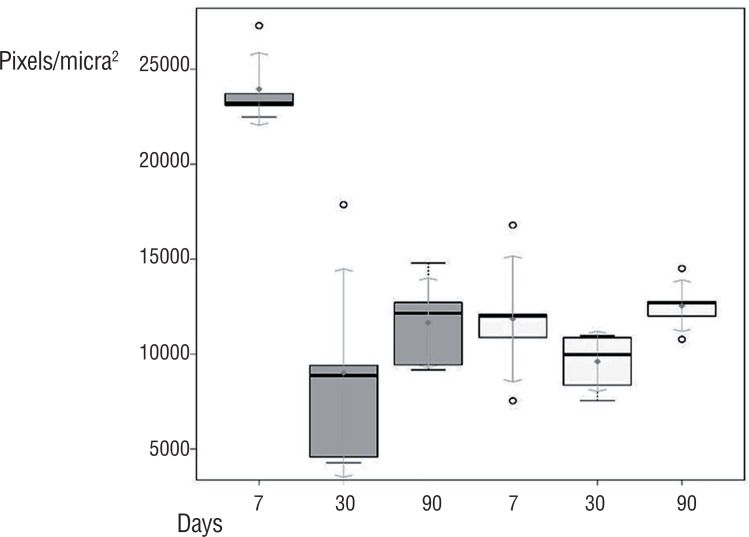
Total collagen boxplots for rabbits sacrificed at 7, 30, and 90 days per group with and without PRP.

**Figure 4:**
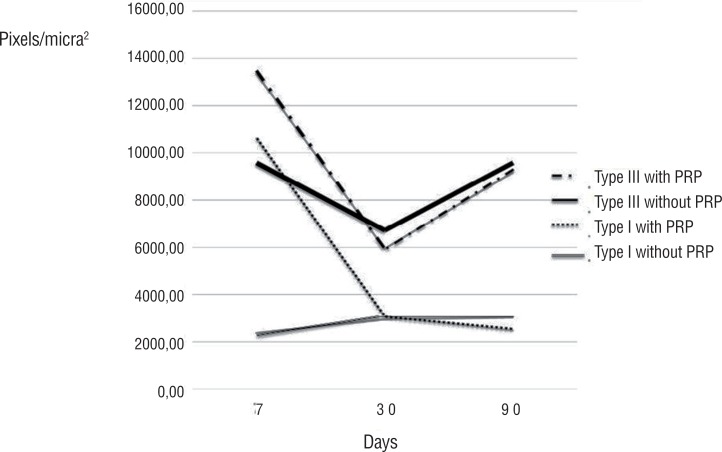
Curve of the average of collagens I and III for the rabbits sacrificed at 7, 30, and 90 days per group with and without PRP.

Figure-3 shows a significant increase of total collagen to rabbits euthanized after seven days for the group with PRP (Wilcoxon test, p=0.008). At seven days, both type I collagen and the type III specimens in the PRP group are shown enlarged (Figure-4 (Wilcoxon test, p=0.001)).

## DISCUSSION

Previous studies have demonstrated that the use of PRP in gynecological surgeries is safe with no apparent side effects (15). Also, using PRP in site-specific prolapse corrections, Gorlero et al. obtained good functional results at 24 months (16). However, in a clinical study with a small number of patients, Einarsson et al. revealed that the PRP application during colporrhaphy does not increase the anatomical results of recurrence (17). These studies confirm that the use of PRP in urogynecology is safe but also suggest the need for animal studies to understand the host's response to implants covered and not covered with PRP (18). Gerullis et al., using mesh coated by plasma in an animal model, concluded that the local inflammatory reaction is an event that occurs immediately after the implantation. It is independent of the implantation site and cannot be influenced by cover plasma.

The data evaluation shows no significant change in inflammatory cells in an initial phase (7 days). This result demonstrates that the PRP does not alter the initial inflammatory response and, therefore, results in no impact at this stage. However, after 30 days, the supply of inflammatory cells starts to increase, reaching a significant difference at ninety days (p=0.01), precisely in the late stage of the process, consolidating the repair. Delavary et al. (19) reported that the inflammatory infiltration and the repair process respond to chemotactic stimuli such as PDGF present in macrophages and platelets. So it is assumed that the continued migration of inflammatory cells favors the end repair probably with macrophages and lymphocytes, which are mainly responsible for maintaining the healing process in the late phase (19).

Our experiment shows an increase mainly of type I collagen when comparing the groups with and without PRP for rabbits sacrificed at seven days, reaching a difference of about 4.5 times. This finding is relevant since this type of collagen is the most common late in the tissue repair process. Therefore, an increase early in the process suggests a possible acceleration in the repair by the action of PRP. However, there was no difference at the late point (90 days), which kept the same level of concentration in individuals who did not undergo PRP use.

The type III collagen also presents an increased production in rabbits euthanized at seven days, but ordinarily, at this early stage, the collagen type III is more active and responsible for tissue repair. Then, the addition of PRP response to the initial increase collagen production can create a better relationship between the mesh and the host. These reflections are evident when integrating the two variable's results: the inflammatory infiltration and collagen production. In an initial process when the intense inflammatory reaction can cause a rejection of the implanted material (20, 21), the PRP did not affect this response. However, when the concentration of inflammatory cells is necessary for the maintenance of the chronic process, providing an adequate material integration, the PRP provides a positive response. Also, the total collagen production increases in the early process, showing a possible acceleration of tissue repair. So it is possible to suggest that a PRP action occurs in the initial phase and the late stage of the process. The PRP acts in different ways but synergistically and probably by the action of growth factors provided by plasma alpha granules.

This study used rabbits in reproductive adulthood with abdominal implants, but the mesh for gynecological use is employed in menopausal women with vaginal implants. Despite the same pathophysiology of healing, abdominal implants occur in sterile conditions differing from the vagina, a potentially contaminated environment.

Since the majority of pelvic reconstructive surgeries are being performed in postmenopausal women, it is essential to understand how estrogen deficiency affects this process.

Another issue is the study times. In a study of animals, Gerullis et al. found that the use of plasma coated mesh did not influence the early inflammatory reaction but did influence the inflammation in the medium and long terms (21). Unfortunately, this experiment is not contemplated in later times to euthanasia, so it is impossible to compare results.

An immunohistochemical evaluation and analysis of the presence of PDGF in the various stages of the process with their comparison to groups with and without PRP at different times can contribute to a better understanding of the subject. Also, the PRP's presence should be evaluated by tests of pro-inflammatory and anti-inflammatory markers in the various stages of the repair process. This strategy can answer questions of the intensity of the inflammatory response necessary for proper mesh integration and if the changes are caused by the PRP's action.

Thus, further studies should be conducted to improve understanding of PRP action in host tissue and its actual validity for use in the clinic.

## CONCLUSIONS

The PPM coating with PRP was associated with increased ININ at the implant area, and an increasing trend during the process of tissue repair. The PPM coated with PRP was related to increased concentration of collagen I, collagen III and the concentration of total collagen increased after seven days of implantation.
